# Eco-Friendly Board from Banana Trunk Residues with Poly(Vinyl Alcohol)/Boric Acid

**DOI:** 10.1021/acsomega.6c01719

**Published:** 2026-04-10

**Authors:** Selim Berk Konca, Aybüke Taşdemir, Mujgan Okur, Fatih Akkurt

**Affiliations:** 37511 Gazi University, Faculty of Engineering, Department of Chemical Engineering, 06570 Ankara, Turkey

## Abstract

The increasing demand for wood-based products and environmental concerns have led to an increasing trend toward raw materials for board production. In this study, boards were produced using banana trunk as filler material and poly­(vinyl alcohol) (PVA)/boric acid (BA) as binder. The effects of varying PVA/BA (1/1–1/3) and filler (DM)/binder­(B) ratios (3/1–6/1) determined as experimental parameters on the physical (density, water absorption, and thickness swelling tests), mechanical (three-point bending tests), flame retardant properties (limiting oxygen index (LOI) analysis), and structural analysis (FTIR, TGA and SEM) of boards were investigated. In the determination of physical properties, it was determined that the densities of the samples were in the range of 790–1770 kg/m^3^ and that the density values generally increased with the increase in the amount of boric acid. Experimental findings showed that the compositions of both binder and filler significantly affected the water absorption and swelling properties of boards. Mechanical test results showed that reducing the PVA ratio (increasing BA) increased the mechanical strength of the boards and that the PVA/BA–1/2 composition had 88% higher strength (48.06 MPa) compared to PVA/BA–3/1. Mechanical strength values for different DM/B plates were determined to be between 5.2 and 15.44 MPa. In addition, very high flame retardancy values were obtained by increasing the boric acid content, with LOI values reaching up to 55. Structural analyses revealed that the thermal and mechanical properties of the boards are a result of the interaction between PVA/BA esterification and the banana trunk. Banana tree trunks, BA, and PVA can be used as sustainable raw materials for the production of environmentally friendly boards with improved mechanical and fire resistance properties.

## Graphical abstract

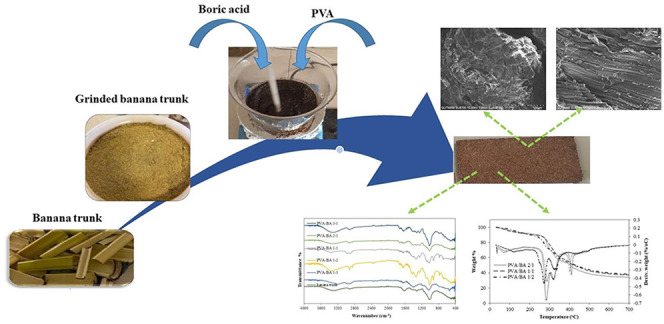

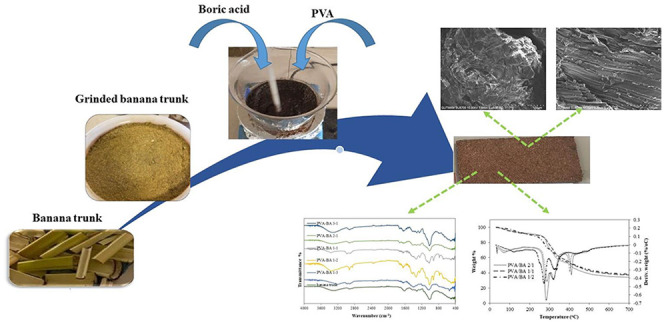

## Introduction

The increasing global population and changes in social structures continuously drive the demand and diversity of wood-based products. To meet this demand, natural resources are being consumed, leading to a decline in the number of these resources. This situation results in a decrease in raw materials and an increase in costs, thereby increasing interest in particleboard production technologies using plant-based and agricultural residues.^1^ To meet the demand for forest products, nearly 100% of each harvested tree (including the trunk, branches, and bark) must be utilized. Today, as awareness of the harmful effects of standard industrial production practices grows, interest in environmentally friendly or green building materials has increased. These materials are nontoxic and produced from renewable or recyclable resources.^2^

Residues from lumber production, low-quality logs, and nonwood agricultural products can be utilized as raw materials in particleboard production.^3,4^ Materials used in particleboard production include a mixture of pine, oak and poplar,^5^ African balsam tree (*Populus balsamifera*) and periwinkle shell residues,^6^ mint fiber-reinforced waste date palm seeds,^7^ waste peach nut shells with glass powder,^8^ oil palm trunk,^3,9,10^ Doum Palm and Balanite tropical fruit shells,^11^ eggplant (*Solanum melongena*) stalks,^12^ Nipah palm fronds,^13^ corn husk (*Zea mays*) and corn silk,^14^ chemically treated fibrous vascular tissue of acai (*Euterpe oleracea* Mart.),^15^ almond shells,^16^ walnut shells,^17^ sycamore leaves and wood particles,^18^ woven banana fiber,^19^ and banana tree wastes.^4,20–24^ Bananas are some of the most important agricultural products globally. The fruit, leaves, flower buds, and stem of the plant are all edible.^25^ Bananas are produced in large quantities in Türkiye and around the world. According to TUİK data, 875,000 tons of bananas were produced in Türkiye in 2024, and according to FAO (Food and Agriculture Organization) data, 139 million tons of bananas were produced in the world. The cellulose (58.5%), hemicellulose (15.4%), and lignin (13.2%) content of banana residues and annual banana production quantities make banana residues a valuable material for various applications.^26^ Beyond its use as a food or dietary supplement, the entire banana plant has potential commercial applications in industries such as packaging and pharmaceuticals.^27^ Additionally, banana stems can be used for making garments and natural handicraft materials.^23^ Research has been conducted on the use of banana trunks in particleboard production.

Currently, growing environmental concerns have led to increased interest in new biobased resins and synthetic binder-free, self-bonding panels as substitutes for formaldehyde-derived binders.^10,28^ Bonding in the absence of binders occurs during the pressing of the particleboard, facilitated by the presence of free sugars, carbohydrates, or saccharides at pressing temperatures.^10^ Various mechanisms have been proposed for self-bonding in lignocellulosic materials, including lignin plasticization, lignin polycondensation, and hemicellulose reactions.^29^ Therefore, for the production of self-bonded and fully lignocellulosic particleboards, various pretreatment techniques such as steam explosion, electromagnetic radiation, chemical pretreatments, or enzymatic pretreatment have been developed to improve the bonding capacity.^30^ For example, Hashim et al. studied the effect of press temperature on the properties of binderless particleboards made from oil palm trunks.^31^ Baskaran et al.^9^ used polyhydroxyalkanoates (PHAs), a completely biodegradable and biocompatible bacterial polyester, to produce binderless particleboards from oil palm trunks and reported that PHAs improved the properties of the particleboards. Similarly, Lamaming et al.^32^ investigated the effects of xylose, sucrose, and glucose additives on binderless particleboards made from young and old oil palm trunks, showing improved particleboard properties. Komariah et al.^33^ demonstrated that ammonium dihydrogen phosphate (ADP) additives improved the physical and mechanical properties of oil palm trunk particleboards. In addition to these studies, there has been research on particleboard production using banana trunks. Jamaludin et al.,^34^ enhanced the properties of binderless particleboards made from banana tree residues through natural lamination materials. They applied poly­(vinyl acetate) on the particleboard surfaces and subjected them to cold pressing at 100 kg/cm^2^ for 24 h. Tests revealed that lamination influenced the modulus of elasticity and modulus of rupture but did not affect internal bonding or water absorption. Baharin et al.^20^ produced particleboards using banana leaves and stalks as raw materials and examined the effect of layer numbers on particleboard properties. Increasing the number of layers improved tensile strength, elongation at break, impact strength, and flexural modulus while reducing elastic modulus. Barragan-Lucas et al.^22^ used urea-formaldehyde resin as a binder for producing particleboards from banana peels. Nunes et al.^24^ prepared banana trunk-based particleboards using cement as a binder and aluminum sulfate (Al_2_(SO_4_)_3_) and sodium silicate (Na_2_SiO_3_) as additives. Ishak et al.^23^ used epoxy resin, while Nadhari et al. and Nongman et al. produced binderless particleboards from banana trunks.^4,21^

In the literature, studies using poly­(vinyl alcohol) (PVA) and boric acid (BA) together in board production are quite limited. In contrast, there are studies where poly­(vinyl alcohol) and boric acid are used together in film or composite production.^35–41^ Boron substances can be used in particleboards in the form of water-soluble solutions at different concentrations. These substances are extremely effective against insects, fungi, and combustion, and are suitable for wood materials above the water level. In a study aimed at examining the effects of boron addition on the physical properties of particleboard, urea-formaldehyde resin was used as a binder along with a hardener and three different boron compounds (borax, boric acid, and a borax-boric acid mixture) in the production of particleboard from softwood and hardwood. It was determined that the specific gravity increased with the addition of boron, except for the boric acid-borax mixture.^42^ In another study examining the properties of particleboards impregnated with zinc borate and boric acid, ammonium chloride was used as a hardening additive. It was observed that the addition of boron (ulexite and colemanite) did not have a negative effect, and colemanite ore was more resistant to biological effects.^43^ Kaya et al.^44^ investigated the effects of boric acid on the properties of fiberboards made from wood/secondary fiber blends. It was observed that the addition of boric acid to the boards had only marginal effects in terms of improving or enhancing the thermal properties. In addition, it has been observed that the plates generally have better acoustic properties at higher frequencies. Poly­(vinyl alcohol) is one of the most important commercially available water-soluble polymers and has a high crystalline structure.^36^ Due to its biodegradability, film-forming, and mechanical properties, poly­(vinyl alcohol) is widely used in packaging materials and many other applications.^45^ Lignocellulosic biomass obtained from various sources was combined with PVA and used in the synthesis of biodegradable composites. In one study, biodegradable composite films with different compositions were prepared using banana pseudostem fiber and a poly­(vinyl alcohol) (PVA) matrix together with plasticizers and cross-linkers via the solution casting method. The effect of the composition on the structure and properties of the resulting films was investigated. Biodegradable composites were prepared using banana pseudostem fiber and poly­(vinyl alcohol), and these composites improved water resistance in films.^45^ In another study, cellulose nanocrystals isolated from bleached banana stem fibers were combined with PVA to prepare nanocomposite films that can be used in packaging materials, and their mechanical, permeability, and optical properties were investigated.^46^ In another study using PVA, the aim was to prepare composites using banana fiber as the main reinforcing fiber and poly­(vinyl alcohol) (PVA) resin as the matrix and to evaluate their mechanical properties. The study reported that the tensile strength of the PVA and banana fiber composite was 18.57 MPa.^47^ Sathasivam et al.^48^ produced poly­(vinyl alcohol)/fatty acid esterified banana stem fibers with various compositions by the solution casting method. In the prepared banana trunk fiber/poly­(vinyl alcohol) blend film structure, it was observed that increasing the amount of banana trunk fiber improved the thermal properties and reduced the swelling degree compared to pure PVA. Although there are studies on particleboard production from banana trunks, no study has been found on board production using banana trunks and PVA/BA as a binder. This study is original in this respect.

From an economic perspective, banana trunks are an abundant agricultural byproduct, resulting in negligible raw material costs. PVA/BA was used as a binder in board production. While PVA-based systems are slightly more expensive than urea-formaldehyde resins, they offer significant advantages such as the absence of formaldehyde emissions, environmental friendliness, and safer processing conditions. Therefore, combining low-cost banana trunks with a formaldehyde-free polymer-based composite binder (PVA/BA) provides a sustainable approach to board production. Similar considerations regarding biomass utilization and sustainable material design have been addressed in previous studies.^49–51^

In this study, to prepare the board, the banana trunk was used as the filling material, and a mixture of poly­(vinyl alcohol) and boric acid was used as the binder. It is aimed to evaluate the effects of PVA/BA and filler/binder ratios on the physical, mechanical, and flame-retardant properties of the boards. Boards were prepared at a 4/1 filler/binder ratio using different ratios of PVA/BA as binder, and the effect of the PVA/BA ratio on the properties of boards was investigated. Additionally, the PVA/BA ratio was fixed at 1:1, while the filler-to-binder ratio was varied between 3:1 and 6:1 to evaluate its impact on board properties. Physical properties of boards, such as water absorption capacity and thickness swelling percentage, were examined. The mechanical properties were assessed using a three-point bending test; flame-retardant properties were evaluated through a limiting oxygen index analysis. The structural properties of the boards were examined by FTIR analysis, thermal analysis by TGA analysis, and the morphological structures by SEM analysis.

## Experimental Method

### Materials

Poly­(vinyl alcohol) (80% hydrolyzed) (CAS No.: 9002-89-5) used in the study with an average molecular weight range of 9000–10,000 was purchased from Sigma-Aldrich. Boric acid (H_3_BO_3_) (99.5–100.5%) (CAS No. 10043-35-3) was supplied by Merck. Banana trunks were harvested from the Antalya region of Türkiye. The harvested trunks were cut into smaller pieces (approximately 10 cm) and dried at room temperature. No other pretreatment was applied to the banana stems used in the studies.

### Preparation of Materials

Banana tree trunks were used as filler material for board production. Since the trunks contain a high amount of water, they were initially cut into smaller pieces (10 cm length and 5 cm width) and allowed to dry at room temperature. Once dried, the banana trunks were ground into smaller particles in a rotary grinder. In experiments where the PVA and BA ratios of PVA/BA used as binders were varied, the amount of banana trunk was used as filler, 50 g, and the total binder amount (PVA + BA) was 12.5 g. The amount of banana trunk used as filler was kept constant at 50 g, while the filler/binder ratio was 4/1, and the PVA/BA ratio (by weight) was changed as shown in Table 1. In experiments where the filler/binder (DM/B) ratio was varied, the filler amount was taken as 50 g, and the binder amount (*B* = PVA/BA ratio 1/1 (w/w)) was taken by weight, as shown in Table 1.

**Table 1:** PVA/BA Ratios (by Weight) Used in The Board Preparation

binder	PVA	BA
PVA/BA-1/1	1	1
PVA/BA-1/2	1	2
PVA/BA-1/3	1	3
PVA/BA-2/1	2	1
PVA/BA-3/1	3	1

filler/binder	filler	binder
DM/B-3/1	3	1
DM/B-4/1	4	1
DM/B-5/1	5	1
DM/B-6/1	6	1

For binder preparation, PVA and boric acid were dissolved separately by heating (80 °C) for 5 h and then mixed to obtain a homogeneous solution at this temperature. The mixing process was carried out in a heated magnetic stirrer. The filler material (banana trunk) was added to the binder in the amounts given in Table 1, and the mixture was mechanically stirred for 15 min. Then, the banana trunk and binder mixture was poured into molds and subjected to pressing. The molds were placed in a press at 130 °C and compressed under 1 t for 45 min. After pressing, the molds were removed from the press and left to cool at room temperature (Figure 1).

**Figure 1:**
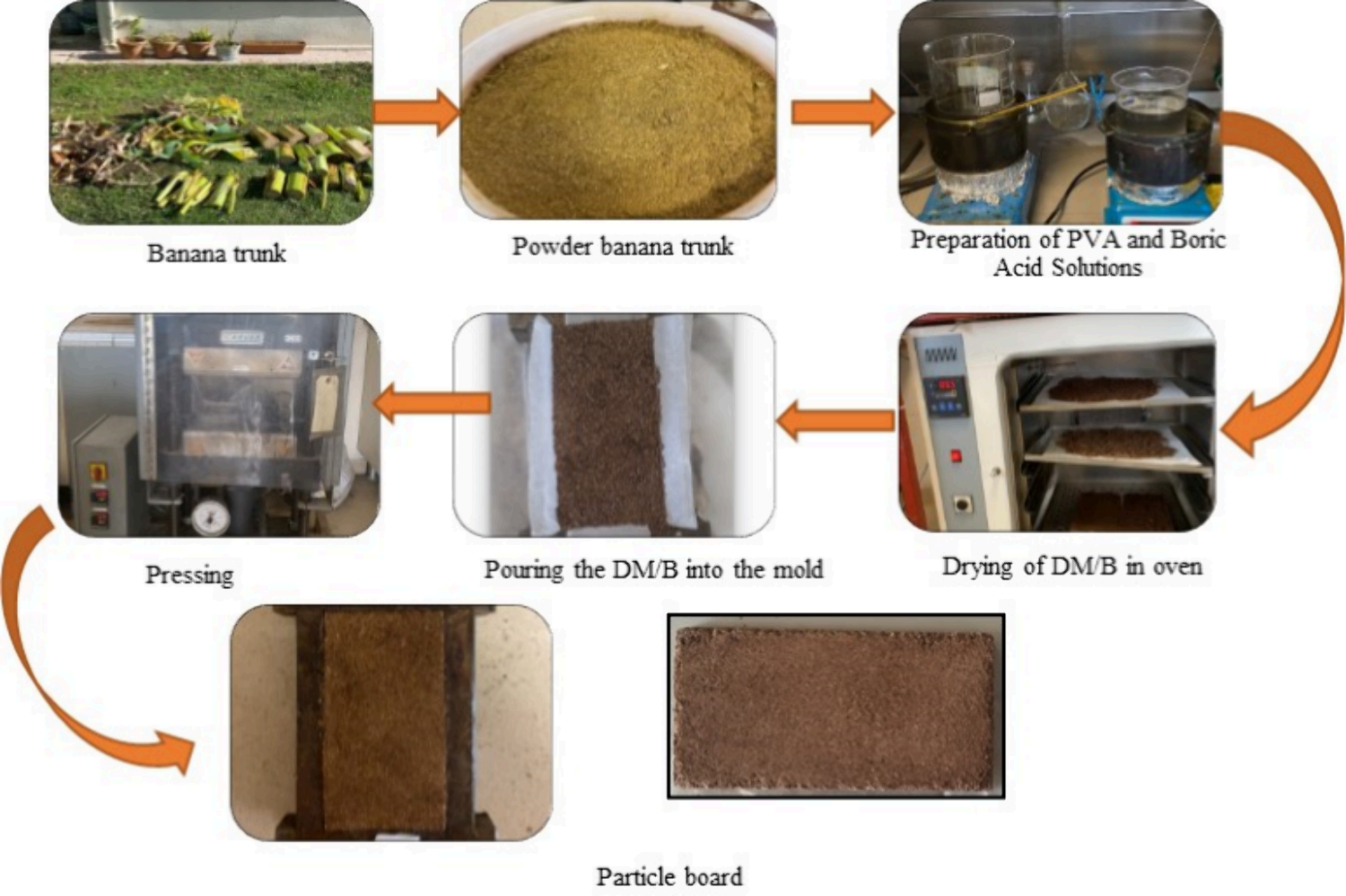
Board production stages.

### Characterization of the Boards

The mechanical and physical properties of the prepared boards were evaluated using density analysis, water absorption tests, mechanical tests, and limited oxygen index (LOI) tests. All analyses were performed in triplicate.

#### Density Analysis

For density analysis, the dry weight of a board piece was first measured. The board was then immersed in a graduated cylinder containing water, and its volume was determined based on the change in the water level. The density analysis was performed in triplicate, and the results were averaged.

#### Water Absorption and Thickness Swelling Tests

Water absorption tests aim to determine the resistance of boards to water. In this test, boards were placed in beakers filled with water to an overflow level. Their weights and thicknesses were measured for 24 h. The water absorption capacity, thickness swelling percentage, and density of the boards were calculated using eqs 1, 2 (ref 8) and 3 (ref 6):

$$\mathrm{Water}\;\mathrm{absorption}\;\mathrm{capacity}\;\left(\frac{g\;\mathrm{water}}{g\;\mathrm{board}}\right)=\frac{\left(m_{\mathrm{wet}}-m_{\mathrm{dry}}\right)}{m_{\mathrm{dry}}}$$ 1

$$\mathrm{Thickness}\;\mathrm{swelling}\;(\%)=\frac{D_{\mathrm{wet}}-D_{\mathrm{dry}}}{D_{\mathrm{dry}}}\times 100$$ 2

$$\mathrm{Density}\;(\frac{\mathrm{kg}}{m^{3}})=\frac{\mathrm{Air}\;\mathrm{dried}\;\mathrm{weight}}{\mathrm{Air}\;\mathrm{dried}\;\mathrm{volume}}$$ 3

where *m*_wet_ is the wet mass of the board at time *t* (g), *m*_dry_ is the initial dry mass of the board (g), *D*_wet_ is the wet thickness of the board at time *t* (cm), *and D*_dry_ is the initial dry thickness of the board (cm).

#### Mechanical Properties

Three-point bending tests were conducted to examine the mechanical strength of the boards. The three-point bending test of the produced materials was determined on a Shimadzu AG-I mechanical testing device in accordance with the EN 310 standard. During the test, the boards were placed between two parallel supports. A compressive apparatus was used to apply force to the boards, measuring their resistance to bending. The data collected from the tests provided the maximum stress and maximum force applied to the surface areas of the boards.

#### Limiting Oxygen Index (LOI) Analysis

The LOI analysis was performed to determine the minimum concentration of oxygen required to sustain combustion of the samples. The test was performed on a Firetesting Technology device at the National Boron Research Institute (BOREN) laboratories. Limiting oxygen index (LOI) values of the boards were determined in accordance with the ASTM D2863 standards. During the test, the board samples were exposed to a flame, and the time taken for combustion to occur was observed. All analyses applied to boards were performed repeatedly. Standard error was indicated in the graphs drawn.

#### FTIR

The structural properties of the boards were investigated using a Jasco 4700 ATR/FT-IR spectrophotometer with a resolution of 4 cm^–1^ and a wavenumber range of 4000–400 cm^–1^.

#### TGA Analysis

Thermal analysis of the boards was performed on an SDT650 brand TGA-DSC device in a nitrogen atmosphere, at a temperature range of 25–700 °C and a heating rate of 10 °C/min.

#### SEM Analysis

The morphological structures of the boards were investigated using FE-SEM analysis with a HITACHI SU8700 FE-SEM instrument.

## Results and Discussion

### Physical Properties of the Boards

The density, water absorption, and thickness swelling characteristics of boards made from banana trunk were analyzed to assess their physical properties. The boards prepared at different PVA/BA ratios and filler material/binder (DM/B) ratios were evaluated for density, water absorption, and thickness swelling properties. It was observed that the boards had smooth surfaces and did not disintegrate after pressing (Figure 2). However, it was noted that the edges of the PVA/BA-3/1 material were cracked and the color darkened (Figure 2). Similarly, the DM/B-5/1 board exhibited a darkened color (Figure 2). The darkening of the color of boards may be due to the thermal decomposition of structures such as cellulose, hemicellulose, and lignin in the banana trunk lignocellulose structure under the effect of heat and pressure during the pressing step. It may also be due to the oxidation of phenolic compounds in the lignin structure. Zhao et al. stated that the chemical structure of sucrose deteriorates at high temperatures, resulting in a decrease in mechanical properties.^52^ Hidalgo-Cordero et al.^29^ stated that the boards they prepared with totora waste changed color during the pressing stage due to surface reactions such as caramelization, and that the color change was associated with an improvement in mechanical properties.

**Figure 2:**
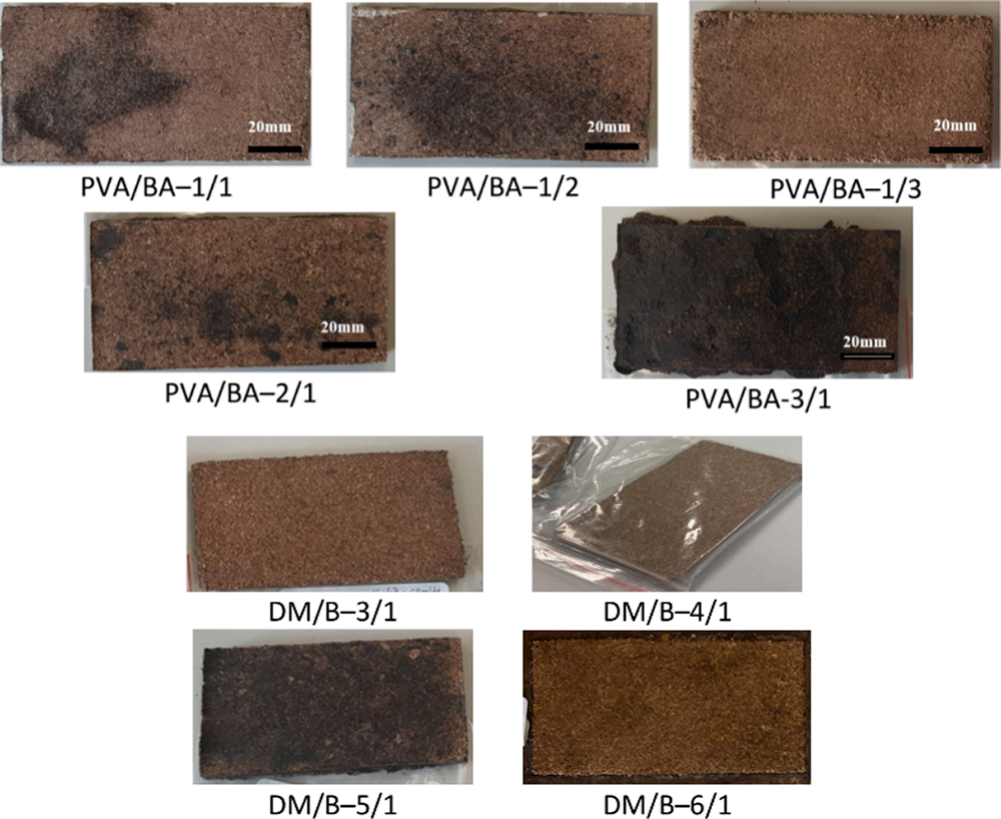
Boards prepared with different PVA/BA and DM/B ratios.

The PVA/BA and DM/B ratios significantly influenced the physical properties of the boards. The bulk densities of the boards were determined and are presented in Figure 3. Density values varied in the ranges of 1130–2770 and 790–1340 kg/m^3^ for PVA/BA and DM/B, respectively. A decrease in the PVA/BA ratio resulted in increased density, but a drop in density was observed at PVA/BA-1/3. The density of the boards is affected by factors such as wood type (wood density), pressing pressure, adhesive content, and other additives.^53^ In this study, an increase in the density of the boards is an expected result with the increase in the amount of boric acid, which has a high density. The PVA/BA–1/2 board has the highest density (1770 kg/m^3^). Although the density of boric acid (∼1500 kg/m^3^) is higher than the density of poly­(vinyl alcohol) (PVA), boric acid alone cannot provide a density increase. The higher density observed in the PVA/BA–1/2 sample may be due to better particle bonding during pressing and fewer internal voids caused by the PVA/BA molecular interaction. In addition, the gel structure formed in the PVA/BA-1/2 ratio, probably through esterification or hydrogen bond interactions between the hydroxyl groups of PVA and borate ions, may have caused the filler particles to be pressed more efficiently, thus reducing porosity. In contrast, the density of the PVA/BA–1/3 board containing even higher boric acid decreased. The higher boric acid in the medium may have negatively affected the homogeneity of the board, increased the formation of voids in the board, and caused the density to decrease. Although no significant effect of the DM/B ratio on bulk density was detected, it was observed that bulk density increased as the binder ratio increased. This outcome is thought to result from the interaction between the filler material and the binder.

**Figure 3:**
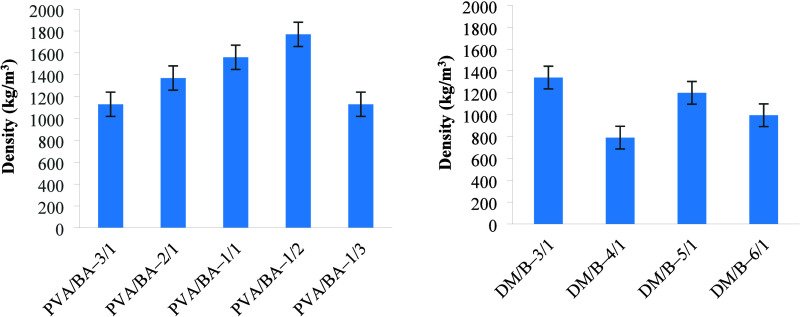
Density of the prepared boards.

The water absorption properties of the samples were investigated for 24 h at room temperature (Figure 4). At the end of 30 min, the PVA/BA–1/3 and PVA/BA–3/1 boards disintegrated, while the PVA/BA–1/1 boards disintegrated after 90 min. The other two boards maintained their structure intact for the full 24-h period without disintegrating. As a result, the final thickness and weight of the disintegrated boards could not be determined. In the first 1.5 h, an increase in boric acid content in the PVA/BA–1/2 and PVA/BA–2/1 boards led to a reduction in both water absorption and thickness swelling. After the 24-h water absorption test, the thickness swelling in PVA/BA–1/2 was 100%, and in PVA/BA–2/1, it was 55.6%. At the end of the water absorption test, PVA/BA-2/1 exhibited a 55.6% increase in thickness and a 137.7% water absorption rate. It is believed that as the PVA ratio increases, the boards become more porous, making them more prone to water absorption. It was observed that the boards with DM/B ratios of 3/1 and 6/1 exhibited very fast water absorption and thickness swelling, with a tendency to disintegrate in the first 60 min of the water absorption test conducted with DM/B. The DM/B–6/1 board, which had the lowest binder amount, showed that the binder was insufficient to hold the filler material together, leading to disintegration during the water absorption test. On the other hand, the DM/B–3/1 board contained more binder than the others, resulting in the filler material being more saturated with water. This caused difficulties during the pressing stage of these boards. The water absorption and thickness swelling properties of the DM/B–4/1 and DM/B–5/1 boards were compared. In the first hour, the board with higher filler material (DM/B–5/1) absorbed less water and exhibited less thickness swelling compared to the other. However, over the 24-h period, DM/B–5/1 had a greater thickness swelling than DM/B–4/1, which had a lower filler content. Similar results were observed in the production of particleboards made from date palm seeds with added mint fiber. Taşdemir et al.^7^ observed that as the filler/polymer ratio increased, the filler material became more porous than the polymeric binder, resulting in higher water absorption capacity. Odeyemi et al.^6^ prepared boards with different proportions of sawdust and sea shells and found that an increase in sawdust (%) and cement (%) led to higher water absorption, while an increase in sea shell content resulted in a reduction in water absorption. Yıldırım and Candan^5^ prepared particleboards with varying proportions of nanocellulose/boric acid and observed changes in their water absorption capacities with varying ratios. In this study, it was also found that as the boric acid content in the boards increased, the water absorption capacity increased. Şahinöz et al.^14^ noted that with an increase in the corn silk fiber content in corn husk/corn silk fiber boards, both swelling and water absorption rates increased. They suggested that the increase in these properties might be due to the creation of voids in the boards by the fibers. These findings suggest that the compositions of both the binder and filler significantly affect the water absorption and swelling properties of boards. The presence of boric acid and changes in the filler-to-binder ratio are key factors influencing the performance of these boards under wet conditions.

**Figure 4:**
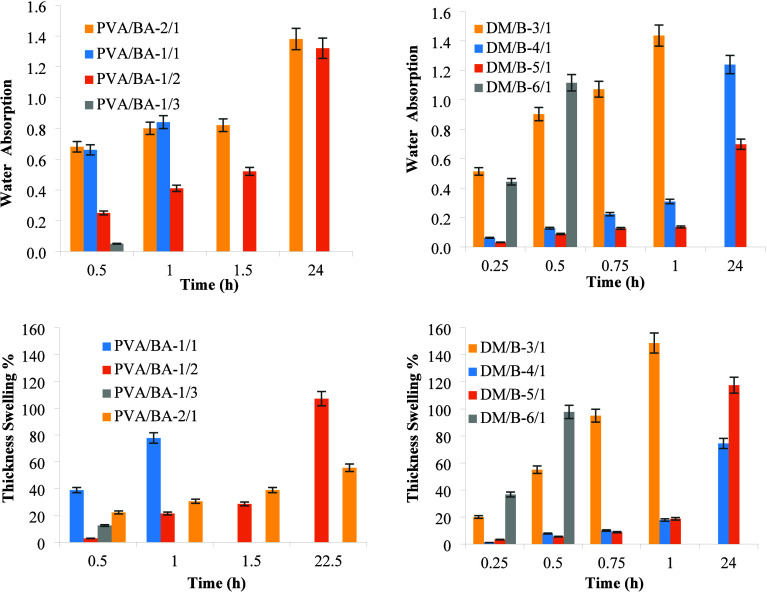
Water absorptions (g water/g board) and thickness swelling % for PVA/BA and DM/B boards.

### Mechanical Properties of the Board

A three-point bending test was applied to assess the mechanical properties of the prepared boards; the maximum force and modulus of rupture (MOR) results for each sample are provided in Figures 5 and 6. As the PVA/BA ratio decreased, both the maximum force and the maximum stress (MOR) values exhibited an increase. However, the rate of increase was lower for the PVA/BA–1/3 boards. The boards with the lowest strength (PVA/BA–1/1, PVA/BA–1/3, and PVA/BA–3/1) also showed lower water absorption resistance. In such boards, the balance between PVA (poly­(vinyl alcohol)) and BA (boric acid) ratios may not be sufficient. If the binder ratio is low or the polymer network structure is not well-formed, the mechanical strength decreases because the fibers cannot be bonded together strongly enough. At the same time, the resistance to water decreases because there are more microcavities or capillary channels inside the structure through which water can leak. As a result, low strength indicates that the binder structure is weak and porous, which means that the resistance to water is low.^54^ According to the analysis, the PVA/BA–1/2 board exhibited the highest mechanical strength with a maximum force of 0.1938 kN and a maximum stress of 48.06 MPa. For the boards with varying filler/binder ratios, the highest MOR was achieved for the DM/B-4/1 board. Based on the analysis, the board prepared with the DM/B–3/1 ratio was determined to be the strongest, with a maximum force of 0.07937 kN and a MOR of 10.14 MPa. It was clearly seen that the strength of the prepared boards increased when the DM/B ratio was changed from 6/1 to 3/1. Binder acts as an adhesive for the filling material and ensures the integrity of the material. When the binder ratio decreases, there is not enough adhesion between the fibers, and weak interface connections are formed, proving that the materials have a stable character within the elastic region, and the situation has led to a decrease in the mechanical properties of the boards. For this reason, the mechanical properties of DM/B-5/1 and DM/B-6/1 boards were low. Taşdemir et al.^7^ stated that as the filler/polymer ratio increased, the mechanical strength value increased from 1.75 to 6.2 MPa. Hashim et al.^31^ stated that the absence of adhesives in boards negatively affects their bending properties, particularly their MOR, especially in binderless boards. There is a linear relationship between the mechanical properties and the density of the boards. Ghani et al.^13^ stated that higher-density boards exhibit superior mechanical properties due to the higher amount of lignin, which helps in the bonding of particles during the pressing process. Similarly, as density increases, MOR tends to increase, while thickness swelling % and water absorption ratio tend to decrease.^22,52^ A similar trend was observed in this study, as well. As the densities of boards containing different ratios of PVA/BA increased, the MOR values also increased. Lamaming et al.^32^ reported that binderless boards were produced from young and old oil palm trunks, and the highest MOR value obtained from the produced boards was 13.56 MPa. Nadhari et al.^4^ obtained a MOR value of 1.54 MPa for binder-free particleboard made from steam-pretreated banana stem waste. The water absorption percentage without steam pretreatment was found to be 246%. Lamaming et al.^55^ reported that the physical and mechanical properties of particleboards prepared using a mixture of modified starch and PVA as an adhesive were improved. Particleboard was produced by Nadhari et al.^4^ using oil palm empty fruit bunch with natural binders such as corn, potato, tapioca, and wheat starch, and the modulus of rupture (MOR) values of the boards were obtained as 15.33 MPa with potato starch, 14.41 MPa with wheat, 14.18 MPa with tapioca, and 12.59 MPa with corn starch.

**Figure 5:**
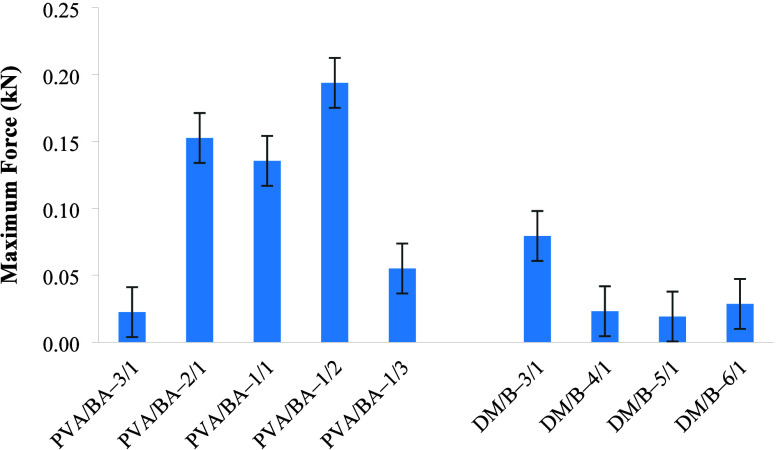
Maximum force values of the boards.

**Figure 6:**
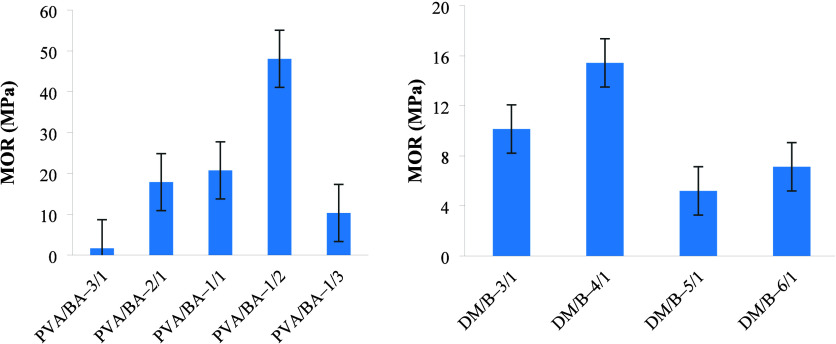
Modulus of rupture (MOR) value for board samples.

Figure 7 shows the force (N)-displacement (mm) relationship of the PVA/BA and DM/B boards. These graphs show that PVA/BA 1/3, PVA/BA 2/1, and PVA/BA 1/2 boards exhibit brittle properties, while the other materials show ductile behavior. On the other hand, the slopes up to the fracture point in the force (N)-displacement (mm) graphs of PVA/BA 1/3 and PVA/BA 3/1 boards are lower than those of the other boards, indicating weaker board properties. These boards were found to have lower densities and lower water resistance compared to the other boards. In the force (N)-displacement (mm) graphs of boards containing different ratios of DM/B, all boards except DM/B-3/1 have lower slopes, and their densities and mechanical strength are lower than those of DM/B-3/1. Furthermore, all boards showed a linear change up to the fracture point.

**Figure 7:**
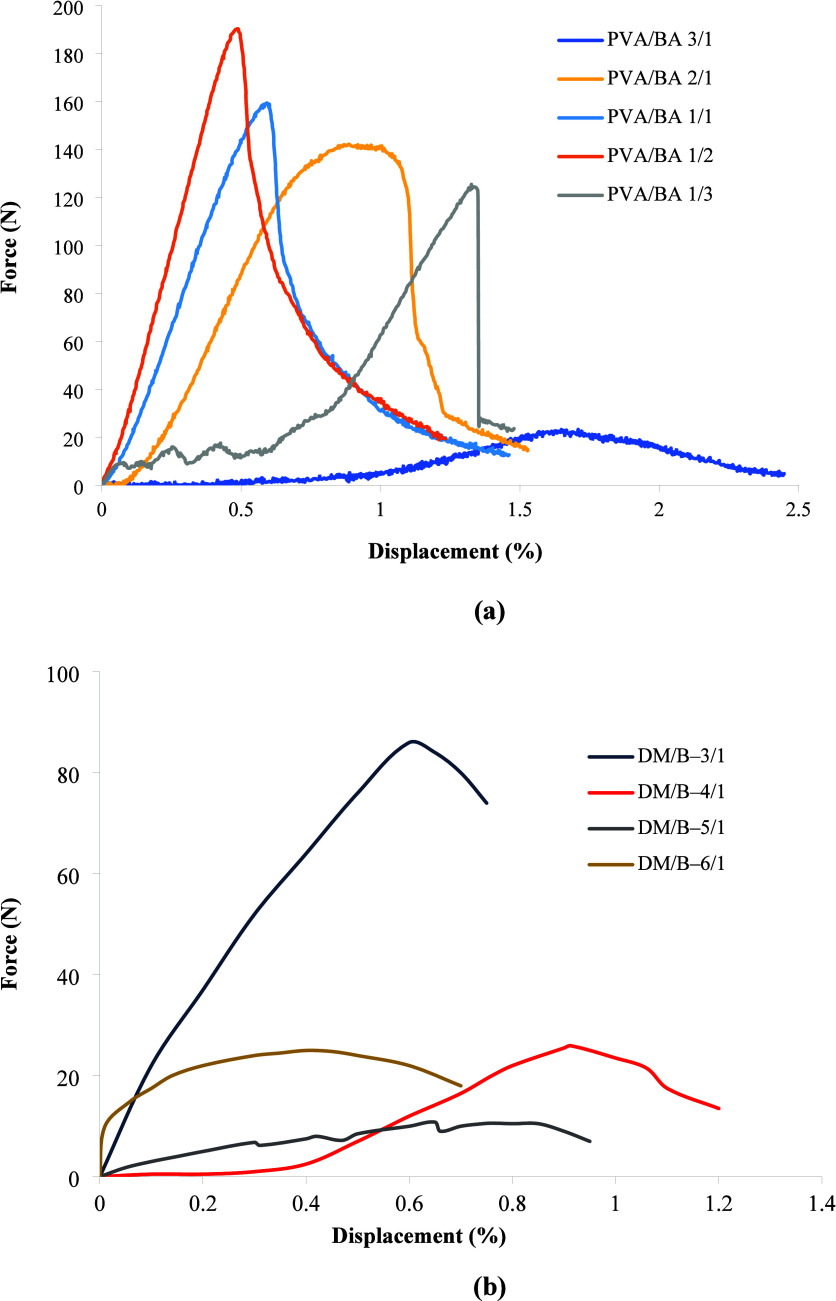
Force (N)-displacement (mm) relationship of PVA/BA (a) and DM/B boards (b).

### Limiting Oxygen Index Test

The Limiting Oxygen Index (LOI) indicates the required oxygen concentration in the atmosphere for combustion. Limiting oxygen index results of PVA/BA and DM/B boards prepared at different ratios are given in Figure 8. According to the LOI test results, the presence of boric acid in the boards was observed to provide flame-retardant properties. For boards containing different PVA/BA ratios, the LOI values increased with the increasing boric acid ratio. The boards prepared with different PVA/BA ratios were found to have LOI values ranging from 30.5 to 55. The highest LOI value was obtained for the PVA/BA–1/2 boards, which ignited when the oxygen level reached 55%. The LOI values for boards prepared with different DM/B ratios ranged from 32.5 to 53. As the filler material ratio increased in DM/B boards, the LOI values decreased. This was due to the reduction in the binder ratio, which led to a decrease in the boric acid content. As a result, the LOI values decreased. Overall, it was observed that the boards maintained their integrity without igniting, demonstrating that the boards could remain flame-resistant under atmospheric conditions. Taşdemir et al.^7^ reported that, similar to the increase in mechanical strength as the filler/polymer ratio increased, increasing the amount of polymer in the structure improved the fire resistance of the material.

**Figure 8:**
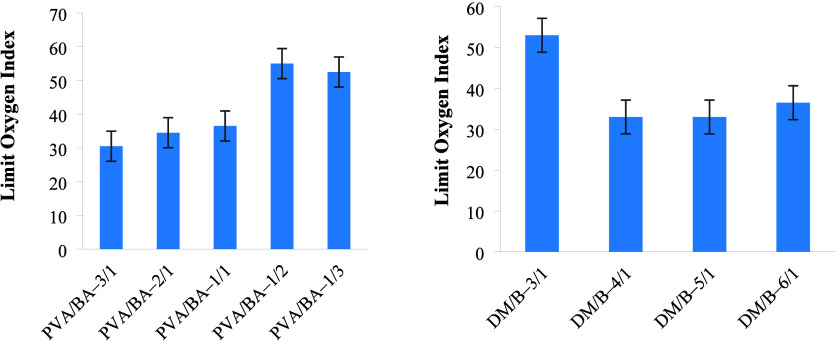
LOI values of the PVA/BA and DM/B boards.

### FTIR Analysis

To examine the structural properties of the boards, the interaction between PVA and BA was investigated first. For this purpose, PVA/BA mixtures with different ratios used in the boards were prepared, and their FTIR spectra were taken before mixing with banana trunk (Figure 9). In these spectra, the broad peak between 3100 and 3600 cm^–1^ in the FTIR spectrum of pure PVA is located at 2938, 2912, 1733, 1424, 1374, 1237, 1088, 1021, 945, and 844 cm^–1^. The broad peak between 3100 and 3600 cm^–1^ in the spectrum of pure PVA indicates OH stretching vibration of the hydroxyl group, while 2938 and 2912 cm^–1^ indicate the asymmetrical and symmetrical CH_2_ stretching vibration.^56,57^ The peaks at 1733 and 1569 cm^–1^ indicate the C=O stretching vibration, and the peak at 1424 cm^–1^ indicates the C–H bending vibration of CH_2_. The peaks at 1088 and 1021 cm^–1^ indicate C–O stretching vibration. The peak at 844 cm^–1^ indicates C–C stretching vibration.^58,59^ The peaks at 1374 and 1237 cm^–1^ correspond to the methyl groups (−CH_3_) or −CH_2_ wagging vibrations, respectively. The peak at 1327 cm^–1^ is attributed to C–H vibrations. The peak at 1141 cm^–1^ corresponds to C–C and C–O–C stretching vibrations; this peak indicates the crystallinity of PVA.^57^

**Figure 9:**
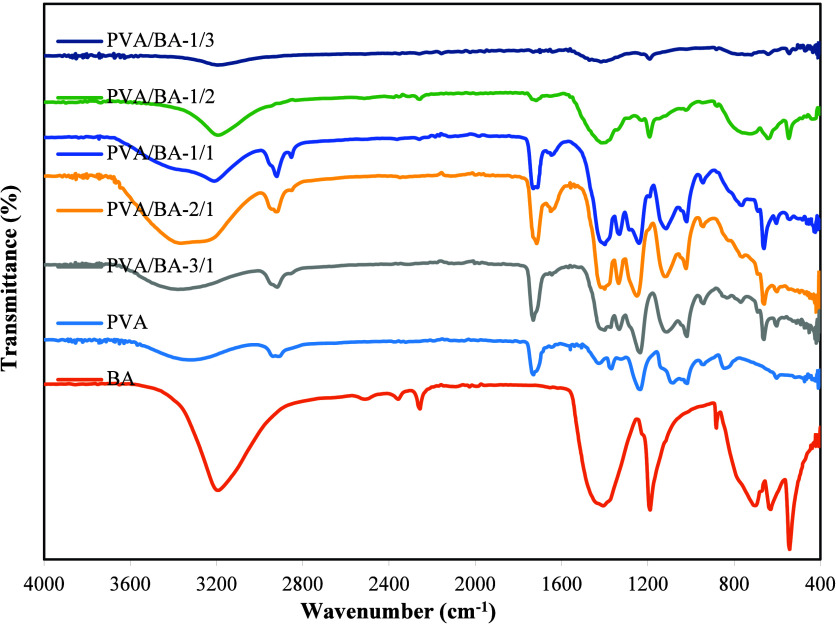
FTIR spectra of BA, PVA, and PVA/BA mixtures at different ratios (no board).

In Figure 9, the peak at 3200 cm^–1^ shows characteristic OH stretching vibrations of pure boric acid,^60,61^ while the peaks at 1400 and 1200 cm^–1^ indicate asymmetric B–O stretching and in-plane B–O–H bending vibrations. The peaks at 884 and 634 cm^–1^ show out-of-plane BO_3_ angle deformation mode, and 544 cm^–1^ corresponds to in-plane O–B–O angle deformation mode.^62^ In PVA/BA mixtures with different ratios, some changes were observed in the spectra as the ratios of PVA and BA changed. In PVA/BA-3/1, 2/1, and 1/1 mixtures, where PVA was present in higher amounts, characteristic peaks belonging to PVA were observed. In contrast, in PVA/BA-1/2 and 1/3 mixtures, where the BA ratio was higher, characteristic peaks belonging to boric acid were observed. Prosanov et al.^39^ stated that the characteristic peaks belonging to PVA remained unchanged in the FTIR spectra of PVA/BA-3/1. However, in the spectra of PVA/BA with different ratios, it was observed that as the boric acid ratio increased, the intensity of the broad peak at 3100–3600 cm^–1^ decreased, and the broad peak shifted toward 3200 cm^–1^ (in the PVA/BA-1/1, 1/2, and 1/3 spectra). Zhang et al.^60^ reported that in PVA/BA films, the peak of BA at 3180 cm^–1^ disappeared in the spectra, and the peak of the O–H group shifted from 3264 cm^–1^ for pure PVA film to 3243 cm^–1^ for PVA/BA-1.0 wt %. However, in the spectra of PVA/BA mixtures, new peaks belonging to the metaboric acid vibration band at 766 cm^–1^ and B–O–B at 662 cm^–1^ emerged.^37,39^ The new peak appearing at 1335 cm^–1^ in the PVA/BA spectra is attributed to the antisymmetric vibration of B–O bonds.^38^ The C=O stretching vibration observed at 1660 cm^–1^ in the PVA/BA structure can be attributed to the boric ester bond.^63^ Furthermore, the BA (in-plane B–O–H bending vibration) peak at 1200 cm^–1^ is not observed in the FTIR spectrum of PVA/BA-3/1, but appears as a very small peak in the others. The complete absence of this peak indicates the absence of unreacted boric acid in the medium.^64^

To examine the functional groups in the board structure and observe the interactions between the board and binder, FTIR spectra were obtained for all boards (Figure 9). Peaks were observed in the FTIR spectra of the pure Banana trunk structure at 3100–3600, 2966, 2918, 2850, 1740, 1637, 1618, 1420, 1364, 1320, 1244, 1146, 1016, and 864 cm^–1^. The broad peak structure observed in the 3100–3600 cm^–1^ wavelength range indicates OH stretching vibration, while the peaks at 2966 and 2918 cm^–1^ show asymmetric and symmetric CH_2_ stretching vibrations.^48,65^ The peak observed at 1740 cm^–1^ indicates the C=O stretching vibration, and the peak at 1637 cm^–1^ indicates the bending mode of the absorbed water^48^ and C=C stretching vibration.^46^ The peaks at 1244 and 1320 cm^–1^ show quaiacyl ring and syringyl ring of the C–O bond present in the lignin structure, and the peak at 1420 cm^–1^ shows the typical bending vibrations of the C–H bond.^48,65^ The stretching vibration of the characteristic C–O–C bond was detected at 1016 cm^–1^.^66,67^ The band at 1160 cm^–1^ corresponds to C–O antisymmetric bridge stretching,^48^ and 864 cm^–1^ indicates C–H deformation in the cellulose and hemicellulose group in the structure of the banana trunk.^46^

In the spectra of boards obtained by mixing banana trunk with binders (PVA/BA 1/1) in different ratios, new peaks were observed, in addition to peaks belonging to the banana trunk structure (Figure 10). New peaks were observed at 1260, 1077, 798, and 661 cm^–1^ in the DM-B board structure. Similarly, new peaks were observed at 782, 924, 1097, 1250, and 1340 cm^–1^ in the FTIR spectra of PVA-BA 1–1 and 1–2 boards containing different ratios of PVA/BA. In the hydroxyl stretching region (3100–3600 cm^–1^), a different absorption band is observed for PVA, BA, and banana trunk. This band shifted depending on the DM/B and PVA/BA ratios when the board was prepared with PVA, BA, and a banana trunk. This may be due to the formation of a new hydrogen bond between PVA and BA or between DM and B. A similar situation was observed in the interaction between PVA and lignin in the study by Korbag et al.^56^ Furthermore, an increase in the intensity of the peaks at 2966 and 2918 cm^–1^ was observed in the spectra of DM/B. In the spectra of the boards, the peaks at 1260 and 1077 cm^–1^ show the wagging vibration of CH_2_ and the stretching vibration of C–O in the PVA structure. The peak observed at 1340 cm^–1^ shows asymmetric stretching vibration relaxation of B–O–C.^57^ In conclusion, the new peaks appearing in the FTIR spectra of the boards indicate that there are some interactions among banana trunk, PVA, and BA.

**Figure 10:**
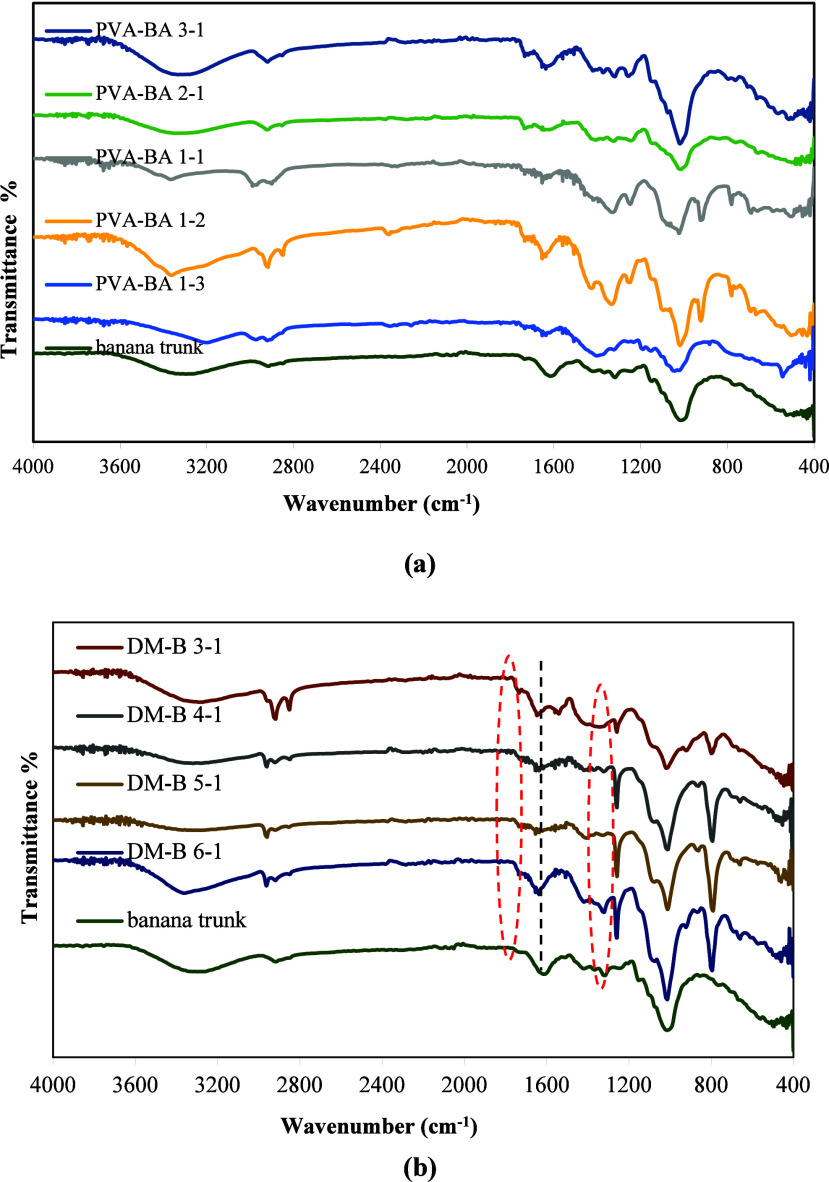
FTIR spectra of boards containing different ratios of PVA/BA (a) and DM-B (b).

### TGA Analysis

Figure 11a shows the weight percentage and derivative weight curves of the boards. The initial mass loss started at 32 °C for PVA/BA 2/1 and PVA/BA 1/1 and at 70 °C for PVA/BA 1/2. This is thought to be due to the water absorbed in the board structure. The initial mass loss continues up to approximately 250 °C. Kumar et al.^68^ suggested that this situation might be due to some low molecular weight volatile compounds from the banana trunk biomass. The prolonged duration of the initial mass loss phase may be due to the decomposition of PVA or BA that did not react during the PVA/BA esterification phase. Sevim et al.^69^ stated that BA dehydrates at 118 °C to form metaboric acid, with a mass loss of up to 28% at this stage, and above 162 °C, it transforms into boric oxide, with a mass loss of up to 43% at this stage. The temperature at which pure PVA first begins to decompose is around 220 °C.^40^

**Figure 11:**
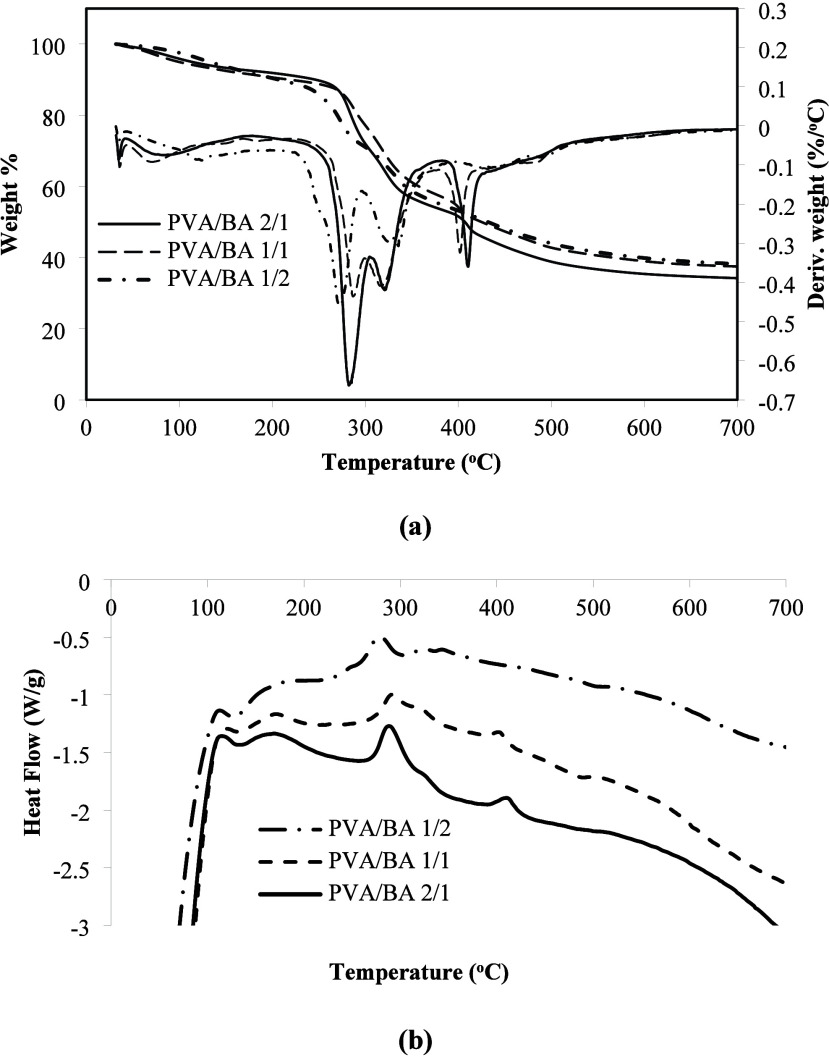
Thermogravimetric analysis of the boards, weight % and Deriv. weight (a) and heat flow (b).

The second mass loss occurred in the 250–300 °C range for all three materials. This mass loss was associated with the thermal decomposition of hemicelluloses, carbon dioxide, and water.^10,31^ Kumar et al.^68^ stated that the second major mass loss for banana trunk biomass occurred in the 150–325 °C range. The initial degradation of hemicellulose and cellulose takes place in this temperature range. An increase in the decomposition temperature of the cellulose/hemicellulose structure was observed with the addition of PVA/BA to the banana trunk. The third mass loss occurred at 400 °C for PVA/BA 2/1 and PVA/BA 1/1. This mass loss at this temperature is attributed to increased lignin degradation and endothermic/exothermic interactions between various degradation products.^68^ Figure 11a also shows the DTG curves of PVA/BA. The DTG curves show that there is a 3-stage decomposition for all three materials, and endothermic peaks corresponding to the decomposition processes are observed. Decomposition peaks were observed at 288, 320, and 410 °C for PVA/BA 2/1 and at 293, 318, and 402 °C for PVA/BA 1/1. Similarly, decomposition peaks were observed at 278 and 324 °C for PVA/BA 1/2. The decomposition temperatures for all three materials were obtained to be quite close to each other. On the other hand, Figure 11b shows the DSC diagram. Peaks were observed in the regions corresponding to the decomposition temperatures in the DSC curves of the boards. These peaks suggest that they may be due to the endothermic nature of the decomposition processes.

### SEM Analysis

SEM analysis (Figure 12) was performed to examine the surface morphologies of the banana trunk biomass and PVA/BA boards. SEM images show banana trunk particles dispersed in different sizes. During the board formation process using banana trunks and PVA/BA, a more integrated structure was formed due to the effect of pressing at high temperature and pressure. It was observed that this structure was formed as a result of the good blending and interaction of the banana trunk structure with PVA/BA esters. Thus, the biomass particles are held together, and a more durable structure is formed. In the PVA/BA 3/1 and PVA/BA 1/3 board structures, most of the biomass particles were covered by the ester structure, while some voids and cracks were also observed on the surface. A similar result was observed in the study by Odeyemi et al.,^6^ in SEM images of cement-bonded particleboard produced from African balsam tree (Populus balsamifera) and periwinkle shell residues. However, in the structures of other boards, it has been observed that banana trunk particles mix very well with PVA/BA, and the particles are completely coated with the binder (PVA/BA 2/1, PVA/BA 1/1, and PVA/BA 1/2). No banana trunk particles, cracks, or voids were observed in these boards. It is thought that this situation improves the mechanical properties of the boards. A similar situation was observed with modified starch and modified starch mixed with poly­(vinyl alcohol) as a binder in particleboard.^55^ Conversely, the voids and cracks in PVA/BA 3/1 and PVA/BA 1/3 board constructions have led to poor mechanical properties in the boards. This also makes the boards more susceptible to water damage, causing them to disintegrate in water.

**Figure 12:**
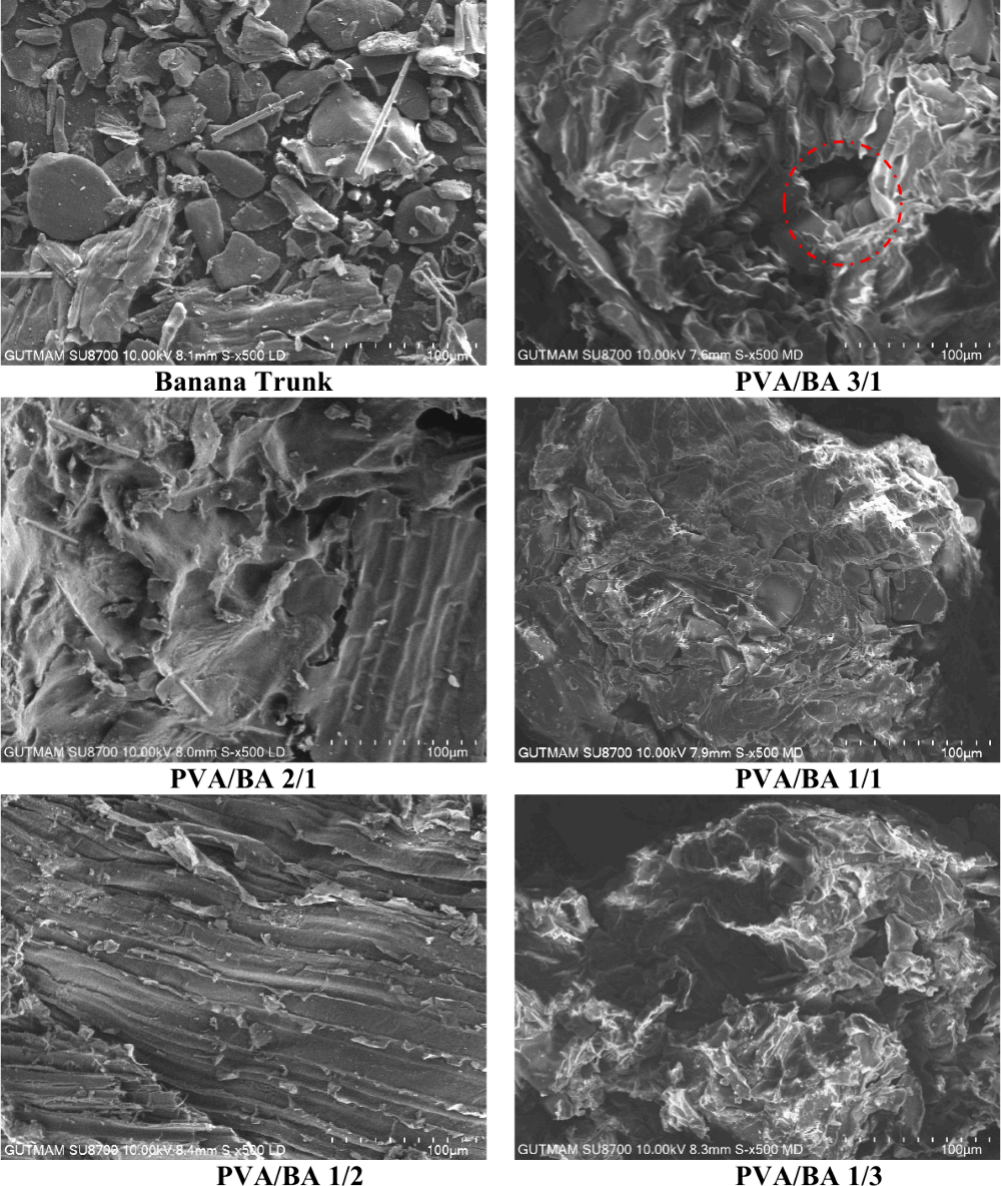
SEM images of the banana trunk and PVA/BA boards.

## Conclusion

In this study, boards were prepared using banana stems as the filler material and PVA/boric acid (PVA/BA) as the binder. The results revealed that both the filler material/binder ratio (DM/B) and the PVA/BA ratio significantly affected the properties of the boards. The mechanical properties of the boards varied with different PVA/BA ratios (5.2–48.06 MPa). It was observed that as the PVA/BA ratio increased, the strength of the boards increased due to esterification of PVA-BA and interaction between the banana trunk and PVA/BA. The highest mechanical strength was found in the PVA/BA–1/2 boards, at 48.06 MPa. Additionally, it was observed that as the amount of boric acid in the PVA/BA ratio increased, the LOI values also increased, enhancing the flame-retardant properties of the boards (LOI: 30.5–55). All of the boards maintained their integrity without igniting, demonstrating that they could remain flame-resistant under atmospheric conditions. FTIR analysis revealed new peaks indicating that PVA/BA esterification and banana trunk-PVA/BA interaction occurred in the prepared boards. Significant changes were observed in the physical, mechanical, and thermal properties of the materials due to the interactions detected in the FTIR analysis. Thermogravimetric analysis showed that the degradation of the boards occurred gradually, leading to an increase in the LOI values. SEM analysis results showed that boards mixed well with PVA/BA and banana trunk fibers exhibited good mechanical and other properties. The results of the analyses applied to all boards in the study were found to be correlated with each other. This suggests that the boards prepared in this study could provide an advantage in daily applications, especially in protecting against fire-related risks. Furthermore, the use of natural plant residues and eco-friendly, nontoxic binders in the preparation of these boards promotes a sustainable environmental approach. This study has shown that banana trunk boards can be made by using PVA/BA as a binder. Further studies are planned to improve the properties of these boards.

## References

[1] Thoemen, H ; Irle, M ; Sernek, M . Wood-Based Panels, An Introduction for Specialists. Brunel University Press, 2010.

[2] Muruganandam L., Ranjitha J., Harshavardhan A. (2016). A review report
on physical and mechanical
properties of particle boards from organic waste. Int. J. ChemTech Res..

[3] Baskaran M., Hashim R., Sulaiman O., Hiziroglu S., Sato M., Sugimoto T. (2015). Optimization of press temperature
and time for binderless particleboard manufactured from oil palm trunk
biomass at different thickness levels. Materials
Today Communications [Internet]..

[4] Nadhari W. N. A. W., Danish M., Nasir M. S. R. M., Geng B. J. (2019). Mechanical properties
and dimensional stability of particleboard fabricated from steam pre-treated
banana trunk waste particles. J. Build. Eng..

[5] Yildirim M., Candan Z. (2020). Particleboard with NC/BA. BioResources..

[6] Odeyemi S. O., Abdulwahab R., Adeniyi A. G., Atoyebi O. D. (2020). Physical and mechanical
properties of cement-bonded particle board produced from African balsam
tree (Populous Balsamifera) and periwinkle shell residues. Results in Engineering [Internet]..

[7] Taşdemir H. M., Şahin A., Karabulut A. F., Gürü M. (2019). Investigation
of the properties of composite material produced from mint fiber added
waste palm kernel. J. Fac. Eng. Arch. Gazi Univ..

[8] Sahin A., Tasdemir H. M., Karabulut A. F., Gürü M. (2017). Mechanical
and Thermal Properties of Particleboard Manufactured from Waste Peachnut
Shell with Glass Powder. Arabian Journal for
Science and Engineering..

[9] Baskaran M., Hashim R., Said N., Raffi S. M., Balakrishnan K., Sudesh K., Sulaiman O., Arai T., Kosugi A., Mori Y., Sugimoto T., Sato M. (2012). Properties of binderless
particleboard from oil palm trunk with addition of polyhydroxyalkanoates. Composites, Part B.

[10] Saari N., Hashim R., Sulaiman O., Hiziroglu S., Sato M., Sugimoto T. (2014). Properties of steam
treated binderless
particleboard made from oil palm trunks. Composites
Part B: Engineering [Internet]..

[11] Betené A. D. O., Ndiwe B., Krishnan G. S., Wedaïna A. G., Manfouo Tchoupmene C., Ngongang Djakou C. B. (2023). Processing of tropical
agro-industrial waste for particleboard manufacture: Dimensional stability
and mechanical performance. Journal of Building
Engineering [Internet]..

[12] Guntekin E., Karakus B. (2008). Feasibility of using
eggplant (Solanum melongena) stalks
in the production of experimental particleboard. Industrial Crops and Products..

[13] Mohd
Ghani R. S., Osman M. S., Abdul Rani A. I. (2024). Exploring
the potential of Nipah palm frond as sustainable raw material for
eco-friendly particleboard production. Cleaner
and Circular Bioeconomy [Internet]..

[14] Şahi̇nöz M. E. L. İ. H., Gürü M.
E. T. İ. N., Yilmaz Aruntaş H. Ü. S.
E. Y. İ. N. (2024). Valorization
of Corn Husk (Zea Mays) and Corn Silk in Polymer Particleboard Manufacture
and Effect of Waste Colemanite on the Mechanical Performance of Particleboards. Cellul. Chem. Technol..

[15] de
Lima Mesquita A., Barrero N. G., Fiorelli J., Christoforo A. L., De Faria L. J. G., Lahr F. A. R. (2018). Eco-particleboard manufactured from
chemically treated fibrous vascular tissue of acai (Euterpe oleracea
Mart.) Fruit: A new alternative for the particleboard industry with
its potential application in civil construction and furniture. Ind. Crops Prod..

[16] Gürü M., Tekeli S., Bilici I. (2006). Manufacturing
of urea-formaldehyde-based
composite particleboard from almond shell. Materials
and Design..

[17] Gürü M., Atar M., Yildirim R. (2008). Production of polymer
matrix composite
particleboard from walnut shell and improvement of its requirements. Materials and Design..

[18] Pirayesh H., Moradpour P., Sepahvand S. (2015). Particleboard from wood particles
and sycamore leaves: Physico-mechanical properties. Engineering in Agriculture, Environment and Food [Internet]..

[19] Sapuan S. M., Leenie A., Harimi M., Beng Y. K. (2006). Mechanical properties
of woven banana fibre reinforced epoxy composites. Materials and Design..

[20] Baharin A., Fattah N. A., Bakar A. A., Ariff Z. M. (2016). Production of Laminated
Natural Fibre Board from Banana Tree Wastes. Procedia Chemistry [Internet]..

[21] Nongman A. F., Baharin A., Bakar A. A. (2016). The Effect of Banana
Leaves Lamination
on the Mechanical Properties of Particle Board Panel. Procedia Chemistry [Internet]..

[22] Barragàn-Lucas A. D., Llerena-Miranda C. R. I. S. T. H. I. A. N., Quijano-Avilés M. F., Chóez-Guaranda I. A., Maldonado-Guerrero L. C., Manzano-Santana P. I. (2019). Effect
of resin content and pressing temperature on
banana pseudostem particle boards properties using full factorial
design. An. Acad. Bras. Cienc..

[23] Ikman
Ishak M., Ismail C. N., Khor C. Y., Rosli M. U., Riduan Jamalludin M., Hazwan M. H. M. (2019). Investigation on the
Mechanical Properties of Banana Trunk Fibre-Reinforced Polymer Composites
for Furniture Making Application. IOP Conf.
Ser.: Mater. Sci. Eng..

[24] Nunes L., Cintura E., Parracha J. L., Fernandes B., Silva V., Faria P. (2021). Cement-bonded particleboards
with
banana pseudostem waste: Physical performance and bio-susceptibility. Infrastructures.

[25] Thandavamoorthy R., Devarajan Y., Kaliappan N. (2023). Antimicrobial, function, and crystalline
analysis on the cellulose fibre extracted from the banana tree trunks. Scientific Reports [Internet]..

[26] Suhani N., Radin Mohamed R. M. S., Abdul Latiff A. A., Nasir N., Ahmad B., Oyekanmi A. A., Awang H., Daud Z. (2020). Removal of COD and
ammoniacal nitrogen by banana trunk fiber with chitosan adsorbent. Malays. J. Fund. Appl. Sci..

[27] Hefnawy A. H. T., Awad A. E., Wahdan K. M., Doheim M. A. (2022). Chemical
and Functional
Properties of the Native Banana (MUSA) Pseudo-Stem. Biotechnology Research..

[28] Nasir M., Khali D. P., Jawaid M., Tahir P. M., Siakeng R., Asim M. (2019). Recent
development in binderless fiber-board fabrication
from agricultural residues: A review. Construction
and Building Materials [Internet]..

[29] Hidalgo-Cordero J. F., García-Ortuño T., García-Navarro J. (2020). Comparison
of binderless boards produced with different tissues of totora (Schoenoplectus
californicus (C.A. Mey) Soják) stems. J. Build. Eng..

[30] Espinosa E., Tarrés Q., Theng D., Delgado-Aguilar M., Rodríguez A., Mutjé P. (2021). Effect of enzymatic treatment (endo-glucanases)
of fiber and mechanical lignocellulose nanofibers addition on physical
and mechanical properties of binderless high-density fiberboards made
from wheat straw. J. Build. Eng..

[31] Hashim R., Said N., Lamaming J., Baskaran M., Sulaiman O., Sato M. (2011). Influence of press temperature
on the properties of
binderless particleboard made from oil palm trunk. Materials and Design [Internet]..

[32] Lamaming J., Hashim R., Sulaiman O., Sugimoto T., Sato M., Hiziroglu S. (2014). Measurement
of some properties of binderless particleboards
made from young and old oil palm trunks. Measurement:
Journal of the International Measurement Confederation [Internet]..

[33] Komariah R. N., Miyamoto T., Tanaka S., Prasetiyo K. W., Syamani F. A., Subyakto, Umezawa T., Kanayama K., Umemura K. (2019). High-performance
binderless particleboard from the inner part of oil palm trunk by
addition of ammonium dihydrogen phosphate. Ind.
Crops Prod..

[34] Jamaludin M. A., Bahari S. A., Zakaria M. N., Azizan U. A. (2020). Improvement of binderless
banana pseudo-stem particleboard properties via natural laminating
materials. Solid State Phenomena..

[35] Chousidis N. (2024). Polyvinyl
alcohol (PVA) -based films: insights from crosslinking and plasticizer
incorporation Polyvinyl alcohol (PVA) -based fi lms: insights from
crosslinking and plasticizer incorporation. Eng. Res. Express..

[36] Gadhave R. V., Mahanwar P. A., Gadekar P. T. (2019). Study of Cross-Linking
between Boric
Acid and Different Types of Polyvinyl Alcohol Adhesive. Open Journal of Polymer Chemistry..

[37] Şen İ. (2025). EFFECT
OF SiO_2_ ADDITION ON MECHANICAL AND THERMAL PROPERTIES OF
BORIC ACID CROSSLINKED POLYVINYL ALCOHOL (PVA) FİLMS. Uludağ University Journal of The Faculty of Engineering..

[38] Lambertini L., Coccarelli G., Toto E., Santonicola M. G., Laurenzi S. (2024). Poly­(vinyl alcohol) gels cross-linked by boric acid
for radiation protection of astronauts. Acta
Astronautica [Internet]..

[39] Prosanov I. Y., Abdulrahman S. T., Thomas S., Bulina N. V., Gerasimov K. B. (2018). Complex
of polyvinyl alcohol with boric acid: Structure and use. Mater. Today Commun..

[40] Fatih
Isik A., San Keskin N. O., Ulcay Y. (2019). Synthesis and in vitro
antimicrobial characterization of Boron-PVA Electrospun nanofibers. Journal of the Textile Institute [Internet]..

[41] Xu X., Xie S., Shi B., Wu D., Lin Y., Zhi Q. (2025). Synergistic
reinforcement of PVA films with boric acid and nano-silica
for high-barrier food packaging. Polym. Testing.

[42] Var A. A. (2012). Influences
of adding rates of the boron compounds on the surface soundness of
particleboards. J. Bartin Faculty Forestry.

[43] Terzi E., Kartal S. N., Gerardin P., Ibanez C. M., Yoshimura T. (2017). Biological
performance of particleboard incorporated with boron minerals. Journal of Forestry Research..

[44] Kaya A., Sahin H. (2017). The Effects of Boric
Acid on Fiberboard Properties Made from Wood/Secondary
Fiber Mixtures: Part 4. Insulation Properties of Boards. Journal of
Applied Life Sciences. International..

[45] Srivastava K. R., Singh M. K., Mishra P. K., Srivastava P. (2019). Pretreatment
of banana pseudostem fibre for green composite packaging film preparation
with polyvinyl alcohol. J. Polym. Res..

[46] Pereira A. L. S., Nascimento D. M. d., Souza Filho M. d. s. M., Morais J. P. S., Vasconcelos N. F., Feitosa J. P. A., Brígida A. I.
S., Rosa M. d. F. (2014). Improvement
of polyvinyl alcohol properties by adding
nanocrystalline cellulose isolated from banana pseudostems. Carbohydr. Polym..

[47] Rana R. S., Rana S., Nigrawal A., Kumar B., Kumar A. (2020). Preparation
and mechanical properties evaluation of polyvinyl alcohol and banana
fibres composite. Materials Today: Proceedings
[Internet]..

[48] Sathasivam K., Mas Haris M. R. H., Noorsal K. (2010). The preparation and characterization
of esterified banana trunk Fibers/Poly­(vinyl alcohol) blend film. Polym.-Plast. Technol. Eng..

[49] Yu Z. L., Ma Z. Y., Yao H. X., Qin B., Gao Y. C., Xia Z. J. (2022). Economical Architected
Foamy Aerogel Coating for Energy
Conservation and Flame Resistance. ACS Materials
Letters..

[50] Zuo, C. ; Hui, X. Y. ; Xin, P. A. ; Qi, H. N. ; Shen, X. ; Wen, J. L. , An integrated biorefinery strategy for dual valorization of xylose residue unlocks high-yield glucose and fully lignin-based adhesives. Chemical Engineering Journal [Internet]. 2026;529(December 2025):172786. Available from: 10.1016/j.cej.2026.172786.

[51] Grzegorzewska E., Burawska-Kupniewska I., Boruszewski P. (2020). Economic profitability of particleboards
production with a diversified raw material structure. Maderas: Cienc. Tecnol..

[52] Zhao Z., Hayashi S., Xu W., Wu Z., Tanaka S., Sun S. (2018). A novel eco-friendly
wood adhesive composed by sucrose
and ammonium dihydrogen phosphate. Polymers..

[53] Iswanto A. H., Sutiawan J., Darwis A., Lubis M. A. R., Pȩdzik M., Rogoziński T., Fatriasari W. (2023). Influence of Isocyanate Content and
Hot-Pressing Temperatures on the Physical–Mechanical Properties
of Particleboard Bonded with a Hybrid Urea–Formaldehyde/Isocyanate
Adhesive. Forests.

[54] Jain N., Singh V. K., Chauhan S. (2017). A review on mechanical and water
absorption properties of polyvinyl alcohol based composites/films. Journal of the Mechanical Behavior of Materials..

[55] Lamaming J., Heng N. B., Owodunni A. A., Lamaming S. Z., Khadir N. K. A., Hashim R. (2020). Characterization of rubberwood particleboard
made using carboxymethyl starch mixed with polyvinyl alcohol as adhesive. Composites, Part B.

[56] Korbag I., Mohamed Saleh S. (2016). Studies on
the formation of intermolecular interactions
and structural characterization of polyvinyl alcohol/lignin film. Int. J. Environ. Stud..

[57] Al-Emam E., Soenen H., Caen J., Janssens K. (2020). Characterization
of
polyvinyl alcohol-borax/agarose (PVA-B/AG) double network hydrogel
utilized for the cleaning of works of art. Heritage
Science [Internet]..

[58] Jabbar W. A., Habubi N. F., Chiad S. S. (2010). Optical Characterization
of Silver
Doped Poly (Vinyl Alcohol) Films. J. Arkansas
Acad. Sci..

[59] Kharazmi A., Faraji N., Mat Hussin R., Saion E., Yunus W. M. M., Behzad K. (2015). Structural, optical,
opto-thermal and thermal properties
of ZnS-PVA nanofluids synthesized through a radiolytic approach. Beilstein J. Nanotechnol..

[60] Zhang S., Wei D., Xu X., Guan Y. (2023). Transparent, High-Strength, and Antimicrobial
Polyvinyl Alcohol/Boric Acid/Poly Hexamethylene Guanidine Hydrochloride
Films. Coatings.

[61] El-Batal A. I., El-Sayyad G. S., Al-Hazmi N. E., Gobara M. (2019). Antibiofilm
and Antimicrobial
Activities of Silver Boron Nanoparticles Synthesized by PVP Polymer
and Gamma Rays Against Urinary Tract Pathogens. Journal of Cluster Science [Internet]..

[62] Attia N., Ahmed H., Yehia D., Hassan M., Zaddin Y. (2017). Novel synthesis
of nanoparticles-based back coating flame-retardant materials for
historic textile fabrics conservation. Journal
of Industrial Textiles..

[63] Wang C., Shen Z., Hu P., Wang T., Zhang X., Liang L. (2022). Facile
fabrication and characterization of high-performance
Borax-PVA hydrogel. Journal of Sol-Gel Science
and Technology..

[64] Gadhave R. V., Vineeth S. K., Dhawale P. V., Gadekar P. T. (2020). Effect
of boric
acid on poly vinyl alcohol- tannin blend and its application as water-based
wood adhesive. Des. Monomers Polym..

[65] Okur M., Eslek Koyuncu D. D. (2020). Investigation
of pretreatment parameters in the delignification
of paddy husks with deep eutectic solvents. Biomass Bioenergy.

[66] Hoang N. T. T., Tran A. T. K., Hoang M. H., Nguyen T. T. H., Bui X. T. (2021). Synergistic
effect of TiO2 /chitosan/glycerol photocatalyst on color and COD removal
from a dyeing and textile secondary effluent. Environmental Technology and Innovation [Internet]..

[67] Nguyen V. V., Tran T. N., Nguyen T. H. (2025). Activated
carbon from banana trunk
fiber as an effective electrode material for desalination via capacitive
deionization. Int. J. Environ. Stud..

[68] Kumar M., Shukla S. K., Upadhyay S. N., Mishra P. K. (2020). Analysis of thermal
degradation of banana (*Musa balbisiana*) trunk biomass
waste using iso-conversional models. Biores.
Technol..

[69] Sevim F., Demir F., Bilen M., Okur H. (2006). Kinetic analysis of
thermal decomposition of boric acid from thermogravimetric data. Korean Journal of Chemical Engineering..

